# The burden of chronic mercury intoxication in artisanal small-scale gold mining in Zimbabwe: data availability and preliminary estimates

**DOI:** 10.1186/1476-069X-13-111

**Published:** 2014-12-13

**Authors:** Nadine Steckling, Stephan Bose-O’Reilly, Paulo Pinheiro, Dietrich Plass, Dennis Shoko, Gustav Drasch, Ludovic Bernaudat, Uwe Siebert, Claudia Hornberg

**Affiliations:** Department Environment & Health, Bielefeld University, School of Public Health, Universitätsstraße 25, D-33615 Bielefeld, Germany; University Hospital Munich, Institute and Outpatient Clinic for Occupational, Social and Environmental Medicine, WHO Collaborating Centre for Occupational Health, Workgroup Paediatric Environmental Epidemiology, Ziemssenstr. 1, D-80336 Munich, Germany; UMIT - University for Health Sciences, Medical Informatics and Technology, Department of Public Health and Health Technology Assessment, Institute of Public Health, Medical Decision Making and Health Technology Assessment, Eduard Wallnoefer Center I, A-6060 Hall i.T., Austria; Bielefeld University, Faculty of Educational Sciences, Universitätsstraße 25, D-33615 Bielefeld, Germany; Federal Environment Agency, Section Exposure Assessment and Environmental Health Indicators, Corrensplatz 1, D-14195 Berlin, Germany; Tailjet Consultancy Services, 4 Tor Road, Vainona, Borrowdale, Harare Zimbabwe; Institute of Forensic Medicine, Department of Forensic Toxicology, University of Munich - LMU, Nussbaumstr. 26, D-80336 Munich, Germany; United Nations Industrial Development Organization, Vienna International Centre, P.O. Box 300, A-1400 Vienna, Austria; Harvard Medical School, Massachusetts General Hospital, Institute for Technology Assessment and Department of Radiology, Boston, USA; Harvard School of Public Health, Department of Health Policy and Management, Center for Health Decision Science, Boston, USA

**Keywords:** Environmental burden of disease, Disability-adjusted life years, Artisanal small-scale gold mining, Mercury, Occupational health, Zimbabwe

## Abstract

**Background:**

Artisanal small-scale gold mining (ASGM) is a poverty-driven activity practiced in over 70 countries worldwide. Zimbabwe is amongst the top ten countries using large quantities of mercury to extract gold from ore. This analysis was performed to check data availability and derive a preliminary estimate of disability-adjusted life years (DALYs) due to mercury use in ASGM in Zimbabwe.

**Methods:**

Cases of chronic mercury intoxication were identified following an algorithm using mercury-related health effects and mercury in human specimens. The sample prevalence amongst miners and controls (surveyed by the United Nations Industrial Development Organization in 2004 and the University of Munich in 2006) was determined and extrapolated to the entire population of Zimbabwe. Further epidemiological and demographic data were taken from the literature and missing data modeled with DisMod II to quantify DALYs using the methods from the Global Burden of Disease (GBD) 2004 update published by the World Health Organization (WHO). While there was no disability weight (DW) available indicating the relative disease severity of chronic mercury intoxication, the DW of a comparable disease was assigned by following the criteria 1) chronic condition, 2) triggered by a substance, and 3) causing similar health symptoms.

**Results:**

Miners showed a sample prevalence of 72% while controls showed no cases of chronic mercury intoxication. Data availability is very limited why it was necessary to model data and make assumptions about the number of exposed population, the definition of chronic mercury intoxication, DW, and epidemiology. If these assumptions hold, the extrapolation would result in around 95,400 DALYs in Zimbabwe’s total population in 2004.

**Conclusions:**

This analysis provides a preliminary quantification of the mercury-related health burden from ASGM based on the limited data available. If the determined assumptions hold, chronic mercury intoxication is likely to have been one of the top 20 hazards for population health in Zimbabwe in 2004 when comparing with more than 130 categories of diseases and injuries quantified in the WHO’s GBD 2004 update. Improving data quality would allow more accurate estimates. However, the results highlight the need to reduce a burden which could be entirely avoided.

**Electronic supplementary material:**

The online version of this article (doi:10.1186/1476-069X-13-111) contains supplementary material, which is available to authorized users.

## Background

Although mercury (Hg) is highly toxic [1], it is used in artisanal small-scale gold mining (ASGM) to extract gold from ore. This is currently the main global source of anthropogenic mercury emissions [2] and affects both the environment and human health [3]. Due to the informal character of ASGM [4], the magnitude of its impact on human health and the extent of the problem have rarely been studied [5–7].

ASGM is a widespread activity [4, 8, 9] practiced in over 70 countries [10]. While Colombia was recently identified as the worldwide largest mercury polluter per capita due to gold mining [11], Zimbabwe is one of the world’s top ten users of mercury in ASGM, with 25 tons per year [10]. In the extraction process, elemental mercury forms an amalgam with the gold in the ore. It is mostly the poor segment of the population and even children who use this procedure to earn money for themselves and their families [4, 8, 9, 12]. Amalgamation is not the only method, but it is very common because it is simple and mercury is cheap and widely available [4, 8, 9]. The main route of mercury exposure in ASGM is inhalation of vaporized elemental mercury released during amalgam smelting [13], although exposure to other forms of mercury and absorption paths cannot be excluded. Mercury is dumped in the environment during mining, increasing the concentration in the atmosphere and international water bodies, where it is converted to methylmercury [8, 10, 14, 15]. Methylated mercury thus affects humans through food (e.g., contaminated fish) and water [13, 16].

In addition to the mercury body burden measurable in human specimens, chronic exposure to mercury vapors causes erethism, tremor, gingivitis, and other symptoms [17, 18]. Already Scopoli, the physician of the Idrija Mercury Mine in Slovenia in the 18^th^ century, described chronic intoxications of mercury miners including symptoms like tremor, sleep disorders, or periodic contractile movements of legs [19]. Due to its slow onset and diffuse symptoms, it is difficult to make an early diagnosis of chronic mercury intoxication [17]. Health and human biomonitoring studies in several countries have linked increased mercury concentrations in miners exposed to mercury and adverse acute and chronic health effects [20–23].

The toxicology of mercury has been studied extensively [1]. Nevertheless, its impact on population health was not addressed as it should have been [24], as by quantifying attributable costs e.g., [25, 26] or the environmental burden of disease (EBD) by considering exposed subgroups e.g., [27]. EBD is the environmental component of the burden of disease (BoD) approach, where certain amounts of disease burden are attributed to environmental risk factors [28]. The BoD approach developed and applied by the World Health Organization (WHO), the World Bank, and the Harvard School of Public Health [29] allows estimating a population’s health status by using the outcome disability-adjusted life years (DALYs). This method, which has increasingly been used in public health to support policy decision-making, combines several dimensions of disease and allows comparing hazards, diseases, years, and populations and offers ways to monitor public health issues [28]. Using this method, Poulin and Gibb [27] focused on prenatal exposure to methylated mercury, mainly through fish consumption. The burden of methylmercury-induced neurodevelopmental effects in infants was quantified. Furthermore, the toxicity of elemental mercury in highly exposed populations, such as artisanal small-scale gold (ASG) miners, is highlighted but could not be quantified yet due to insufficient data. Recently, the Blacksmith Institute has found use of mercury in ASGM to be the worst of the top ten toxic pollution problems, measured using the estimated number of people at risk. Around 3.5 million people at 135 sites are estimated to be at risk, while 10–20 million people worldwide are believed to work in ASGM. Based on these findings, the Blacksmith Institute first attempted to quantify the global health burden of mercury in ASGM using the EBD approach. This, however, failed because of a lack of data needed to quantify DALYs [30].

Although there is a growing public health awareness of the importance of mercury in ASGM, no comprehensive and comparative assessment of the impact on human health is available. While the Blacksmith Institute could not quantify the global burden, our study provides a preliminary analysis at a national level. The objective of the analysis was to check the current state of research and data availability for a first estimate of the DALYs due to chronic mercury intoxication following occupational mercury use in ASGM in Zimbabwe in 2004 by using the methods from the Global Burden of Disease and Injury (GBD) 2004 update published by the WHO [31]. This allows a comparison with the leading causes of disease burden in Zimbabwe as determined in the GBD 2004 update.

## Methods

The starting point for the DALY estimation was a comprehensive literature search. The main sources were articles listed by PubMed which were supplemented with reports from international organizations involved in ASGM research (e.g., WHO, International Labor Organization [ILO], United Nations Environment Programme [UNEP]). The main key search terms were artisanal, small-scale, gold, mining, mercury, chronic, intoxication, poisoning, mercurialism, Zimbabwe, Africa, environmental, global, burden of disease, and disability weight. The single terms were combined to form search strings.

A case definition for chronic mercury intoxication was formulated before primary data from two projects in Zimbabwe were analyzed to determine the sample prevalence of chronic mercury intoxication (Formula 1, Table 1). The exposure prevalence for all of Zimbabwe was determined (Formula 2, Table 1) and used to extrapolate the sample disease prevalence to the total population (Formula 3, Table 1). Further data needed for DALY estimation (Formulas 4 and 5, Table 1) were compiled and processed. In addition to the main analysis, scenario analyses of prevalence determination and DALY quantification were done to establish the scenario range. The assumptions included in the analysis are summarized in Table 2.

**Table 1:** **Formulas of the analysis**

Formula 1 - Disease point prevalence in the sample (%)	**P**_**i**_ = **D**_**i**_**/E**_i_*100
	P = Prevalence (in %) of the disease in the sample
	D = Number of diseased cases in the sample
	E = Number of individuals in the sample
	_i_ = Age and sex group category i
Formula 2 - Exposure point prevalence in the total population (%)	**EP**_**i**_ **= X**_**i**_ **/T**_**i**_ *100
	EP_i_ = Prevalence (in %) of the exposure in the total population
	X_i_ = Number of exposed individuals in the total population
	T_i_ = Total population
	_i_ = Age and sex group category i
Formula 3 - Disease point prevalence in the total population (%)	**P**_**2**_ **= X**_**i**_ /100*P_**i**_
	P_2_ = Prevalence (in %) of the disease in the total population
	X = Number of exposed individuals in the total population
	P = Prevalence (in %) of the disease in the sample
	_i_ = Age and sex group category i
Formula 4 - Disability-adjusted life years (DALYs)	**DALY**_**i**_ **= YLL**_**i**_ **(a**_**i**_ *b_**i**_ *c_**i**_ **) + YLD**_**i**_ **(d**_**i**_ *e_**i**_ **)**
	DALY = Disability-adjusted life years
	YLD = Years lived with disability
	a = Number of incident cases
	b = Disability weight
	c = Average duration of disability
	YLL = Years of life lost due to premature mortality
	d = Number of deaths
	e = Standard life expectancy at the age of death
	_i_ = Age and sex group category i
Formula 5 - Disability-adjusted life years (DALYs) per 1,000 population	**DALYr**_**i**_ **= DALY**_**i**_ **/T**_**i**_ *1,000
	DALYr = DALYs per 1,000 population
	DALY = Number of total DALYs
	T = Total population
	_i_ = Age and sex group category i

**Table 2:** **Underlying assumptions for the analysis**

a.	Artisanal small-scale gold miners are considered to have an ongoing occupational exposure to mercury.
b.	The diagnostic tool by Drasch et al. [32] was suited to identify cases of chronic mercury intoxication.
c.	The health burden quantified only included the health endpoint “chronic mercury intoxication” (diagnosed according to Drasch et al. [32]) and excluded all other health effects.
d.	The numbers and distribution of the age and sex subgroups of actively involved miners are assumed to be representative of the ASGM sector in Zimbabwe in 2004.
e.	The prevalence of chronic mercury intoxication observed in the sample from Kadoma is representative of all of Zimbabwe in the year 2004.
f.	The proportion of panners and smelter workers in the sample from Kadoma district is representative of the entire population of Zimbabwe.
g.	The modeled data (incidence, duration of disease, etc.) are representative of the disease situation in Zimbabwe.
h.	The severity of chronic mercury intoxication is comparable to the severity of alcoholism, which justifies using the same disability weight.
i.	The severity of chronic mercury intoxication excludes mortality as a consequence.
j.	The severity of chronic mercury intoxication excludes remissions.
k.	The miners included in the study had not received medical treatment for their condition.
l.	The life expectancy of the miners is assumed to be 80 years for males and 82.5 years for females as given in the standard life expectancy table (Standard West Level 26 life table; [28, 29, 31, 33]) used for the Global Burden of Disease (GBD) 2004 update.

The results of this analysis rest on these assumptions. Changing the assumptions will require changing the analysis.

### Case definition

Subjects of the analysis were ASG miners living and working at mining sites in Zimbabwe in 2004 who were actively involved in mercury use and have an ongoing occupational exposure (Assumption a, Table 2). The miners comprised panners and smelter workers. Panners use mercury for amalgamation but do not smelt the amalgam, as is done by smelter workers.

While inhalation of mercury vapors during work might be the most important exposure pathway [13], other exposures such as skin contact or contaminated food or water were not excluded. The mercury body burden was considered without distinguishing between routes of exposure or forms of mercury. Male and female adults as well as children were included in the study. In order to quantify the health burden attributable to mercury use in ASGM, the health outcome “chronic mercury intoxication” as defined by Drasch et al. [32] was chosen (Assumption b, Table 2).

Using an ASGM study from the Philippines, Drasch et al. [32] developed a diagnostic tool for identifying cases of chronic mercury intoxication (Table 3). This diagnosis was part of the protocol for health assessment in the Global Mercury Project conducted by the United Nations Industrial Development Organization (UNIDO) [14]. The UNEP and the WHO included the protocol and the diagnosis in their guidance to identify populations at risk of mercury exposure and strongly encourage using this guidance [13].

**Table 3:** **Diagnosis of chronic mercury intoxication (adapted from**[32]**)**

**[a] Diagnostic algorithm**	**Exposure limit values [b]**	**Medical score sum [c]: Low (0-4)**	**Medical score sum [c]: Mediun (5-9)**	**Medical score sum [c]: High (10-21)**	
	Hg in all biomonitors <HBM I	no intoxication	no intoxication	no intoxication	
	Hg in at least one biomonitor >HBM I and <HMB II	no intoxication	no intoxication	intoxication	
	Hg in at least one biomonitor >HBM II and <BAT	no intoxication	intoxication	intoxication	
	Hg in at least one biomonitor >BAT	intoxication	intoxication	intoxication	
[b] Exposure limit values* for mercury in human biomonitoring samples	**Exposure limit values**	**Hg in blood (μg/l)**	**Hg in urine (μg/l)**	**Hg in urine (μg/g crea.)**	**Hg in hair (μg/g)**
	<HBM I	0-<5	0-<7	0-<5	0-<1
	>HBM I and <HBM II	5-<15	7-<25	5-<20	1-<5
	>HBM II and < BAT	15-<25	25-<30	20-<25	≥5
	>BAT	≥25	≥30	≥25	
**c] Indicators of the medical score sum**	**Anamnesis**		**Clinical examination**	**Neuropsychological tests**	
(0/1): 0= negative; 1= positive; (0/1/2): 0= good, 1= restricted, 2= poor performance; maximum medical score sum: 21 points	Metallic taste (0/1)		Bluish coloring of the gingiva (0/1)	Frostig test^1^ (0/1/2)	
	Excessive salivation (0/1)		Ataxia of gait (0/1)	Matchboxtest^2^ (0/1/2)	
	Tremor at work (0/1)		Finger-to-nose tremor (0/1)	Memory test^3^ (0/1/2)	
	Sleeping problems at night (0/1)		Dysdiadochokinesia (0/1)	Pencil tapping test^4^ (0/1/2)	
	Health problems worsened since		Heel-to-knee ataxia (0/1)		
	having been exposed to		Heel-to-knee tremor (0/1)		
	mercury (0/1)		Mento-labialreflex (0/1)		
			Proteinuria (0/1)		

^*^HBM I and II for blood and urine [34–36]; BAT for blood and urine [37]. HBM I for hair derived by Drasch et al. [32] from the U.S. EPA [38] benchmark limit. HBM II for hair derived by Drasch et al. [32] from the HBM II value for blood [34–36] in combination with results from the Seychelles study [39].

^1^Frostig test for examining tremor and visual-motor capacity: The test person must draw a straight line from one symbol to another across a narrow gap without touching the surrounding areas or breaking the line. This is a subtest of a more detailed test by Lockowandt [40].

^2^Matchbox test for examining coordination, intentional tremor and concentration: The test person must collect matches and put them in the matchbox as quickly as possible while alternating hand. The matchbox test is part of the MOT (Motoriktest) test battery developed by Zimmer and Volkamer [41].

^3^Memory test for short-term memory: The test subject must repeat numbers shown in columns in the correct order. This test was developed by Masur [42].

^4^Pencil tapping test for examining intentional tremor and coordination: The test person must make as many dots as possible in 10 seconds by repeatedly tapping a pencil on a piece of paper. This test is also part of the MOT battery [41].

BAT: Biologischer Arbeitsplatztoleranzwert, the maximum acceptable concentration at the workplace; crea.: creatinine; HBM I and II: Human Biomonitoring values from the Human Biomonitoring Commission of the Federal Environmental Agency; Hg: mercury.

The conclusion of the ASGM study in the Philippines was that higher mercury concentrations in human biomonitors alone do not warrant a diagnosis of chronic mercury intoxication [32]. The biomonitors do not adequately indicate the mercury burden in tissues, particularly the brain [14]. Therefore, a diagnostic algorithm combining health indicators of chronic exposure and increased mercury concentrations in blood, urine, and hair samples was developed, given that the higher the mercury concentration in the samples, the fewer characteristic symptoms are needed for a positive diagnosis and vice-versa [32]. The combination of the human biomonitoring and health data identified the cases of chronic mercury intoxication (Table 3a).

Mercury values in human biomonitors are compared with exposure limit values (Table 3b). The German Human Biomonitoring (HBM) values, termed HBM I and HBM II [34–36], are “health-related biological exposure limit values” [36]. Mercury concentrations below HBM I are assumed to have no causal adverse health effects, but a health hazard cannot be ruled out for concentrations between HBM I and HBM II. The HBM II level is defined as an intervention level because of the greater risk of adverse health effects [43]. Although these mercury exposure limit values were determined in another setting, UNIDO refers to the German HBM values for categorizing mercury concentrations in human specimens determined in ASGM studies [14].

The German HBM commission defined exposure limit values for mercury in blood and urine but not for hair. Drasch et al. [32] used a benchmark limit developed by the U.S. EPA for mercury in hair as the HBM I level [38]. They also derived an HBM II level for mercury in hair based on the HBM II level for blood together with results from the Seychelles study [39]. They applied a stable ratio of 1:300 of mercury in blood and hair and considered the mercury concentration in hair where adverse effects were visible in the Seychelles study (5 μg/g). The HBM II value for mercury concentrations in hair thus derived is limited by the fact that the population of the Seychelles study was exposed to methylmercury while the ASG miners are mainly exposed to mercury vapor [32].

Besides HBM values, the BAT (Biologischer Arbeitsplatztoleranzwert, biological workplace tolerance level) value for organic and inorganic mercury is considered. The BAT value was derived by the German Research Foundation and determines the highest acceptable concentrations of substances at the workplace [37]. Concentrations below the BAT value usually do not affect the health of employees under workplace conditions [44]. The focus of occupational values like the BAT is on healthy adult workers, not the general population [45], which explains the higher exposure limit value compared to HBM values.

It should be considered that mercury concentrations above these exposure limit values do not predict health effects. By definition, health effects cannot be excluded or a greater health risk is present if the exposure limit values are exceeded [43, 45]. The individual susceptibility – influenced by genetic predisposition, physical constitution, lifestyle factors, etc. – is why a clear-cut distinction between hazardous and non-hazardous exposure cannot be made [44]. Based on this fact, these exposure limit values are combined with health data to diagnose chronic mercury intoxication.

The second part of the algorithm for diagnosing chronic mercury intoxication concerns health effects possibly attributable to mercury, summarized in a medical score sum. Table 3c shows the health parameters included. The score was classified as low (0–4), medium (5–9), or high (10–21). It was based on the Philippines study [32] to ensure comparability of results [5].

This case definition excludes other occupationally related health impacts on gold miners from the analysis, such as respiratory outcomes of acute exposure to mercury [46], exposure to other metals [47], or injuries [9] (Assumption c, Table 2).

### Case identification and determination of the sample prevalence

Data from the Global Mercury Project (GMP), conducted by UNIDO in Zimbabwe in 2004 with funds from the GEF (Global Environment Facility) [5, 12], and data from a health and biomonitoring project focusing on women of child-bearing age and their infants, conducted by the University of Munich (LMU; Germany) in Zimbabwe in 2006 [48], were combined to determine the point prevalence of chronic mercury intoxication in occupationally exposed miners and controls (Formula 1, Table 1). A separate analysis of the subgroups panners and smelter workers was not done due to the small subgroup size.

Data on gold miners for both projects were collected in Kadoma. The control group was surveyed in Chikwaka, more than 100 kilometers away from the mining region. Blinded examination was not possible due to the distance between the two study groups [5]. However, the investigators were blinded in terms of the exposure groups (panners, smelter workers) during the medical examination within the mining site. The voluntary participants were selected by the national project manager together with the national and international health experts. All participants gave written consent. The national committee approved the health assessment which was based on extensive legal, formal, and ethical considerations. The Zimbabwean government approved the conditions of the projects. Data collection was done in close collaboration with UNIDO, UNDP (United Nations Development Programme) and the WHO [5, 12, 48].

Mercury concentrations in human specimens – on-the-spot urine, hair, and blood (blood was not collected in 2006) – were compared with HBM I, HBM II, and BAT exposure limit values (Table 3b). The medical score sum was determined using anamnestic data (compiled using questionnaires), clinical examinations, and neuropsychological tests (Table 3c). A binary decision (chronic mercury intoxication: yes/no) was made based on the combination of biomonitoring exposure limit values and the medical score sum (Table 3a).

The primary data were analyzed using SPSS® 16.0. The distribution of sociodemographic characteristics in controls and occupationally exposed subjects was analyzed. To compare controls and exposed, the Chi square test was used to test for interdependence of confounding factors (dental amalgam fillings, alcohol consumption, smoking, consumption of fish, use of retorts, cyanide, skin-lightening creams, confounding diseases, storing mercury and/or working clothes at home, previous mercury exposure), exposure limit values, and cases of intoxication in the subgroups. Details on the collection of human specimens and health data, laboratory analysis procedure, and the medical score sum were published previously [5, 32, 48].

### Extrapolating the disease prevalence to all of Zimbabwe

The percentage of the people exposed in the total population (Formula 2, Table 1) was determined using general population data [49] and published estimates of the numbers of the occupationally exposed population as well as their sex and age distributions. The data necessary for this analysis were obtained from papers listed in PubMed and reports [8, 9, 14, 50–55]. Data and information with the same reference year as this analysis – 2004 – were chosen where available. Furthermore, the sex and age distributions of the 2004 GMP sample randomly collected in Kadoma district also were considered. The data on the age and sex distribution of the miners were assumed to be representative of Zimbabwe (Assumption d, Table 2).

The literature search revealed the following assumptions about the numbers as well as sex and age distributions of occupationally exposed individuals in Zimbabwe around 2004 (Table 4, columns C and D). An estimated 350,000 ASG miners were actively involved in mining and occupationally exposed to mercury [8, 9, 50, 51]. The distinction between adults (85%) and child workers aged 9–14 (15%; 4% girls, 11% boys) is based on the GMP 2004 data. Children ≤8 years old were assumed not to be occupationally exposed. The age distribution of adult miners also was taken from the GMP 2004 project, while representativeness was confirmed by comparing with the age structure reported by Mtetwa and Shava [52]. The same structure was assumed for males and females. Assumptions about the sex distribution in adults were taken from the literature – range of 11% [52, 53] to 50% women [9] – and cross-checked with the GMP 2004 data (21% women). In the main analysis, a mid-level value of the reported data (30% women) was used while the range was included in the scenario analysis.

**Table 4:** **Sample prevalence and assumed distribution of exposed miners in Zimbabwe around 2004**

Column A	B		C		D	E			
Subgroups of artisanal small-scale gold (ASG) miners	Sample prevalence of chronic mercury intoxication in ASG miners^*^		Main analysis: Numbers and percentages^**^of ASG miners assumed for Zimbabwe		Sources of numbers and percentages marked in bold in column C	Scenario analysis: numbers and percentage distribution assumed for Zimbabwe (scenario range)			
						Min.	Source	Max.	Source
**Total (T)**	72%	(131/181)	**350,000**	100%	[8, 9, 50, 51]	No variation		500,000	[54, 55]
**Adults (A)’**	72%	(118/164)	297,500	**85% of T**	GMP 2004	No variation		98% of T	[14]
Males (M)	90%	(95/106)	208,250	**70% of A**	Mid-value of reported data***	50% of A	[8, 9, 50]	89% of A	[52, 53]
15-24	91%	(41/45)	74,350	**36% of M**	GMP 2004 cross-checked against Mtetwa and Shava [52]	No variation			
25-34	88%	(35/40)	86,000	**41% of M**					
35-41	90%	(19/21)	21,450	**10% of M**					
42+			26,450	**13% of M**					
Females (F)	40%	(23/58)	89,250	**30% of A**	Mid-value of reported data***	11% of A	[52, 53]	50% of A	[8, 9, 50]
15-24	26%	(7/27)	31,860	**36% of F**	GMP 2004 cross-checked against Mtetwa and Shava [52]	No variation			
25-34	52%	(16/31)	36,860	**41% of F**					
35-41			9,190	**10% of F**					
42+			11,340	**13% of F**					
**Children”**	76%	(13/17)	52,500	**15% of T**	GMP 2004	2% of T	[14]	No variation	
Male (M)			38,500	**11% of T**					
Female (F)			14,000	**4% of T**					

^*^The data were taken from the Global Mercury Project (GMP), conducted by UNIDO in Zimbabwe in 2004 [5, 12], and from a health and biomonitoring project focusing on women of child-bearing age and their infants, conducted in Zimbabwe in 2006 by the University of Munich (LMU; Germany) [48]. The sample prevalence of the control group is 0%. The sample prevalence (in %) in every subgroup is determined using Formula 1, Table 1. The number of intoxicated and the subgroup size are shown between parentheses.

^**^Numbers and percentages are rounded and include uncertainties because their distribution was derived using the estimates given in the sources in column D.

^***^References consulted with estimates for Zimbabwe [8, 9, 50, 52, 53] cross-checked against the GMP 2004.

^‘^Adults: individuals 15 years and older.

^”^Children: individuals aged 9–14; children aged 0–8 were not assumed to be occupationally exposed.

A: adults; F: females; GMP 2004: own analysis using data from the Global Mercury Project 2004 for Zimbabwe; M: males; Max.: maximum; Min.: minimum; T: total.

For the next step, the sample prevalence of the exposed group and controls (not exposed) was assumed to be representative (Assumption e, Table 2) and was extrapolated to the exposed and unexposed population of the entire country (Formula 3, Table 1). The assumption is reasonable because an estimated 10% of all gold obtained from ASGM in Zimbabwe in 2004 came from the Kadoma-Chakari area [56], which is considered to be a typical ASGM area in Zimbabwe [5]. Furthermore, the proportion of panners and smelter workers in the sample is assumed to be the same across Zimbabwe (Assumption f, Table 2).

### Data needed for estimating disability-adjusted life years (DALYs)

The burden of chronic mercury intoxication in ASGM in Zimbabwe was estimated using the incidence-based DALY approach. DALYs are a combination of years lived with disability (YLD) and years of life lost due to premature mortality (YLL) (Formula 4, Table 1). The YLD component comprises the number of incident cases (a) multiplied by a disability weight (DW) (b) and the average duration of disability (c). The YLL part is calculated by multiplying the number of deaths (d) with a standard remaining life expectancy at the age of death (e). Also, age weights (f) and a discount rate (g) applied for lost life years in the future are included to ensure comparability with the WHO GBD 2004 update [31]. The DALYs calculated were related to the total population of Zimbabwe and presented as rates (DALYs/1,000 population) (Formula 5, Table 1).

### Incident cases (a)

Because no study has estimated the incidence of chronic mercury intoxication in Zimbabwe using the diagnostic tool described above [32], the quantified and extrapolated prevalence of chronic mercury intoxication (see above) combined with additional data (remission and mortality, see below) were used to model the incidence using DisMod II.

DisMod II is a disease-modeling tool developed by the WHO for BoD studies and is based on the logical relations between epidemiological variables. The model allows calculating internally consistent estimates of missing disease-specific data using available variables. The model comprises incidence, prevalence, duration, mortality, relative risk of mortality, case fatality, and remission of the disease. In order to model missing data, information about three of the variables listed is needed. When collecting missing data is not an option, DisMod II can be used to supplement partially available epidemiological data [57]. The data modeled with DisMod II are assumed to be suitable for the analysis (Assumption g, Table 2).

### Disability weight (b)

Calculating the YLD requires a DW indicating the relative disease severity ranging from 0 (perfect health) to 1 (death). Since currently available DW sets e.g., [29, 58, 59] do not include a specific weight for chronic mercury intoxication, a DW from a comparable condition had to be found and used as a proxy. The following set of criteria describing chronic mercury intoxication was developed by the authors and compared with diseases for which DWs were available. A comparable health state should: 1) be a chronic condition, 2) be triggered by a substance, and 3) cause symptoms similar to those of chronic mercury intoxication.

Alcoholism fits the criteria for a comparable DW because it is a chronic condition, it is caused by a substance, and it induces neuropsychological symptoms similar to those of chronic mercury intoxication, such as tremor [14, 32, 60, 61] (Assumption h, Table 2). The DW for alcohol dependence (ICD-10 code F10.2, DW 0.18) [29] was chosen as the best proxy while the DW for harmful use of alcohol (ICD-10 F10.1; DW 0.134) – a second category of alcohol-related disorders [31] – was included in the scenario analysis.

### Duration of disease (c)

The duration of a disease is defined as the time in years between incidence and either remission or death [57]. Since there is no epidemiological data on the duration of chronic mercury intoxication, duration was modeled with DisMod II using available parameters.

### Number of deaths (d)

Information about the case fatality of chronic mercury intoxication was taken from the literature. Cragle et al. [62] did not find a higher mortality amongst workers exposed to elemental mercury vapor compared to unexposed workers, and concluded that mortality is not suitable for assessing chronic exposure to mercury. Case fatality from chronic mercury intoxication was therefore assumed to be zero (Assumption i, Table 2).

Using the case fatality as input data, the number of deaths – internally consistent with the other input data – was modeled using DisMod II.

### Completing the DisMod II input: Remission of disease

In order to model the missing data needed for DALY estimation using DisMod II, three types of input data were needed. In addition to the prevalence (based on epidemiological data) and the case fatality (based on information from the literature), the remission was assumed based on the literature search.

No (zero) remission from chronic mercury intoxication was assumed because mercury damages the central nervous system and, once destroyed, neurons cannot fully regenerate ([63], based on [64]). Depending on the duration and magnitude of exposure, the symptoms will be irreversible, although at least partial remission seems possible in less severe cases [18, 65–68].

The following studies support the assumption of no remission: Follow-up studies showed that neurological symptoms were still seen years or even decades after exposure to mercury had ended [69–80]. The results of these studies are of limited value regarding ASGM because they did not focus on gold miners but on other groups of workers exposed to mercury. Furthermore, the initial exposure of the workers in the other studies was higher than that of the currently observed cohort of gold miners.

The 21 former miners excluded in this analysis were analyzed previously with the result that chronic mercury intoxication was diagnosed in 5 of them [5]. In an ASGM study in Kalimantan, Indonesia, 4 of 10 former miners were diagnosed as intoxicated [81]. Both results support the assumption of persistent health problems, although the analyses are limited by the small sample sizes. Further indications come from a study on former gold miners in Brazil. Possibly mercury-related health symptoms like tremor were more frequent in individuals with high mercury urine concentrations (above 50 μg/l), but clear correlations were not shown and the importance of confounding factors was uncertain [82].

The evidence for assuming no remission of chronic mercury intoxication is weak, but remission cannot be assumed based on the studies cited here. The assumption of ongoing exposure (Assumption a, Table 2) supports the assumption of no remission (Assumption j, Table 2).

Another important factor for remission is treatment [83]. Studies have shown that remission due to improvement of symptoms or a reduction of the mercury body burden appears possible if chelating agents are used [5, 84]. Since therapy is expensive [5], it is unlikely that chelating agents are being used in the study region (Assumption k, Table 2). Hence, including zero remission cases might be reasonable at the current stage of research and follows the assumption that intoxication is severe, as irreversible damage has occurred (Assumption j, Table 2).

### Basic data for DisMod II

Using DisMod II for data modeling, demographic information about the entire population (12,492,000 inhabitants; 6,067,000 male, 43% ≤15 years; 6,425,000 female, 40% ≤15 years [49]) and data on mortality from all causes in Zimbabwe (age-specific death rates) [85] were obtained from the United Nations (UN) and the WHO.

### Life expectancy (e), age weights (f), and discount rate (g)

For this study, the Standard West Level 26 life table (Assumption l, Table 2), a 3% discount rate, and non-uniform age weights [28, 29, 31, 33] were used to ensure comparability with the WHO GBD DALY estimates for 2004 [31].

### Scenario analyses

Scenario analyses were performed for (I) disease prevalence extrapolation for the entire population of Zimbabwe and (II) DALY calculation to observe result variations depending on different scenarios. The sample prevalence as quantified on the basis of the available primary data was not varied in the scenario analysis because there were no conflicting data.

(I) The prevalence extrapolated for chronic mercury intoxication includes estimates and assumptions about the number as well as the sex and age distributions of ASG miners in Zimbabwe. Different sources report different numbers, a fact which was taken into account in the scenario analysis. For the total number of gold miners in Zimbabwe in 2004, the number mentioned most frequently in the literature was used for the main analysis; a less frequently mentioned estimate was included in the scenario analysis. The adult sex ratio varies over a wide range. A mid-value of the reported data was included in the main analysis while the minimum and maximum values were included in the scenario analysis. Assumptions about sex and age distribution of children were taken from the data of the GMP in Zimbabwe in 2004. An estimate of the global involvement of children in ASGM as reported in the literature was included as a scenario.

The conflicting assumptions about number, sex, and age distribution of ASG miners in Zimbabwe were combined in every possible way to determine the extent of result variation. The resulting minimum and maximum estimate of the extrapolated prevalence were used for DisMod II.

(II) Contradictory data were included in scenario analysis II to observe result variation of the DALY calculation (see scenarios 1 to 8 in Additional file 1). Minimum and maximum estimates of the extrapolated overall prevalence of chronic mercury intoxication in Zimbabwe (see above, I) were used for DisMod II to observe the range of variation of the modeled data (scenarios 1 and 2). Furthermore, instead of using the case fatality as a parameter, mortality and the relative risk of mortality were used for DisMod II (scenarios 3 and 4). In order to describe result sensitivity by varying the variable “duration of disease”, it was included with ±10% of the modeled level in the main analysis (scenarios 5 and 6). Furthermore, age weights and discount rates were omitted following ethical considerations [33, 86] and the specifications of the GBD study conducted in 2010 [87] (scenarios 7). Also, another DW was applied (scenarios 8). All variants described for the scenario analyses were examined for subgroups (sex, age) and applied to the main analysis without changing any of the other parameters.

## Results

The partial results of the analyses are described in detail and given in the sections on primary data, extrapolation and modeling, DALY quantification, and scenario analysis.

### Analysis of primary data

#### Characteristics of the sample

Of the initial 403 subjects, 131 were excluded either because of incomplete data (n = 11), acute alcohol intoxication at time of examination (n = 8), a confounding disease (n = 2: brain injury during a car accident, suspected Parkinson’s disease), being a former miner living in an ASGM area (n = 21) or currently living in a control area (n = 2), or being a resident at a mining site without occupational involvement in mining (n = 87). The remaining 272 were included in the analysis. The sample, which included more males than females, comprised 181 miners and 91 controls (Additional file 2). 33 miners were panners who worked in mines and used mercury during amalgamation but did not smelt. Another 148 miners were smelter workers. The overall age range was 9–75 years. The occupationally exposed group had an exposure range of 1–23 years (median: 3 years).

There were several factors which might have confounded the mercury body burden or health effects. From the occupationally exposed group, only 6% (n = 11) used instruments (retorts) to capture mercury vapors. Cyanide was used by 41% (n = 64) of the miners surveyed in 2004 (missing in the 2006 sample). Storing mercury at home (84%; n = 152) and keeping their working clothes at home (98%, n = 177) was typical for the occupationally exposed group. A few individuals in the control and in the exposed group used skin-lightening creams (4% [n = 4] and 6% [n = 11] in the control resp. exposed group; Chi^2^ p = 0.655). None had dental amalgam fillings. The subgroups differed significantly in smoking and drinking habits (both p < 0.001); both were more prevalent amongst miners (20% [n = 36] resp. 33% [n = 60]) than controls (2% [n = 2] resp. 7% [n = 6]). Finally, weekly fish consumption was common amongst miners (68%, n = 122) but not amongst controls (18%, n = 16; p < 0.001).

### Human biomonitoring concentrations and categorizing in exposure limit values

Mercury concentrations in all human biomonitoring samples were considerably higher in the occupationally exposed than in the control group (Additional file 3). The miners showed median and maximum concentrations of 26 and 1,530 μg/l in urine (26 and 667 μg/g creatinine), 11 and 101 μg/l in blood, and 3 and 112 μg/g in hair, whereas controls showed maximum concentrations of 9 μg/l in urine, 2 μg/l in blood, and 3 μg/g in hair. The 95^th^ percentiles for maximum concentration in urine (377 μg/l) imply some outliers with excessively high concentrations in the occupationally exposed. Controls rarely exceeded exposure limit values whereas 80-90% of the miners showed mercury concentrations above exposure limit values, depending on the kind of specimen.

### Health effects of project sample

Findings of possible mercury-induced health effects were seen in both controls and miners. Miners showed the most health effects, with nearly 90% exceeding a medical score sum of 4 points (5–9 points: 72%; 10–21 points: 17%) compared to less than 60% in controls (5–9 points: 57%; 10–21 points: 2%). Detailed analyses of the health effects in the control and the exposed group are given in the Additional file 4.

### Sample prevalence of chronic mercury intoxication

Columns A and B in Table 4 shows prevalence in the sample subgroups. None of the controls showed chronic mercury intoxication while the occupationally exposed showed a prevalence of 72% (131 of 181), albeit with subgroup differences. Prevalence amongst men was similar across age groups, with the youngest (15–24 years) showing the highest prevalence (91%). Older women (≥25 years) showed a prevalence twice as high (52%) as younger women (15–24 years). Prevalence amongst children aged 9–14 (76%) was higher than amongst women. Additional file 4 gives detailed information on the numbers of miners and controls and the diagnostic group they were assigned to depending on the medical score sum and exposure limit values.

### Extrapolating the sample prevalence to all of Zimbabwe

According to the determinants of the analysis, nearly 3% of the total population of Zimbabwe is assumed to have been exposed to occupational mercury use in ASGM in 2004 (Table 5). Of the total population, 2% of the females and 4% of the males were exposed, with more adults exposed than children. The greatest exposure (10%) was seen in males aged 25–34.

**Table 5:** **Extrapolated percentages of exposed and intoxicated miners in Zimbabwe**

Sex and age groups		Total population [[49]]	Main analysis^*^		Scenario analysis	
					Minimum scenario^**^	Maximum scenario^***^
			% exposed of the total population	% of the total population showing intoxication		
Male and female	Total	12,492,000	3	2	2	3
	0-8	3,069,000	(−)	(−)	(−)	(−)
	9-14	2,080,000	3	2	1	1
	15+	7,343,000	4	3	3	6
Male	Total	6,067,000	4	4	3	7
	0-8	1,540,000	(−)	(−)	(−)	(−)
	9-14	1,042,000	4	3	1	1
	15+	3,485,000	6	5	4	11
	15-24	1,533,000	5	4	4	9
	25-34	871,000	10	9	7	18
	35-41	293,000		7	6	14
	42+	788,000	3	3	3	6
Female	Total	6,425,000	2	1	1	1
	0-8	1,529,000	(−)	(−)	(−)	(−)
	9-14	1,038,000	1	1	1	1
	15+	3,858,000	2	1	2	1
	15-24	1,552,000	2	1	1	1
	25-34	870,000	4	2	4	1
	35-41	353,000	3	1	3	1
	42+	1,083,000	1	1	1	1

All numbers and percentages are rounded.

^*^The main analysis includes the numbers given in Table 4 (Column C).

^**^The minimum scenario means that the lowest total prevalence is reached. Included are the following numbers: 350,000 occupationally exposed; 50% of the adults are female; 2% of the total are children (see Table 4 for sources).

^***^The maximum scenario means that the highest total prevalence is reached. Included are the following numbers: 500,000 occupationally exposed; 11% of the adults are female; 2% of the total are children (see Table 4 for sources). In the maximum scenario, there is a low disease prevalence of women and children compared to the minimum scenario, because of the higher sample prevalence of adults compared to children and men vs. women (Table 4). Hence, the maximum scenario is reached when the lowest involvement of children and women is assumed.

(-) children aged 0–8 were not assumed to be occupationally exposed.

The prevalence of chronic mercury intoxication attributable to occupational use in Zimbabwe was estimated at 2% of the total population (scenario range 2-3%). Prevalence in men ranged from 3% (9–14 and 42+ years) to 9% (25–34 years), and in women from 1% (all age groups except 25-34 years) to 2% (25–34 years). Nearly 2% of the children (9–14 years) were assumed to show chronic mercury intoxication from mercury use in ASGM in Zimbabwe in 2004, with boys showing a prevalence three times higher than girls (nearly 3% compared to 1%).

### Modeled data

Table 6 shows the modeled incidence cases, mortality cases, and duration of disease. No mortality was initially assumed, but 4 deaths caused by chronic mercury intoxication based on Zimbabwe’s total population were modeled with DisMod II using the input data to create an internally consistent output. Incidence was modeled with a total of >17,500 cases (males and females) while young males were affected most heavily (around 7,000 cases in the age group 15–24 years). For males and females, an average duration of chronic mercury intoxication of 35 years was modeled.

**Table 6:** **Modeled epidemiological data, YLL and YLD for sex and age groups (main analysis)**

Age^*^(years)	Chronic mercury intoxication				
	Death cases^**^	YLL	Incident cases^**^	Duration of disease (years)^**^	YLD^#^
Occupationally exposed males (n = 246,750; see Table 4)					
**0-8**	<1	<10	200	44	1200
**9-14**	<1	<10	4,900	40	28,900
**15-24**	1	20	7,000	35	38,200
**25-34**	<1	10	2,300	26	10,100
**35-41**	<1	<10	<100	21	<100
**≥42**	1	20	<100	15	<100
**Total**	2	50	14,400	(average) 35	78,400
Occupationally exposed females (n = 103,250; see Table 4)					
**0-8**	<1	<10	<100	48	500
**9-14**	<1	<10	600	43	3,400
**15-24**	<1	<10	1,200	36	6,700
**25-34**	<1	<10	1,400	29	6,400
**35-41**	<1	<10	<100	26	<100
**≥42**	1	20	<100	20	<100
**Total**	2	30	3,300	(average) 35	17,000

All numbers are rounded.

^*^Age structure according to Table 4.

^**^Modeled using DisMod II.

^**#**^YLD calculation includes a 3% discount rate and non-uniform age weights. The Standard West Level 26 life table was used according to the Global Burden of Disease Study [28, 29, 31, 33].

YLL: years of life lost due to premature mortality; YLD: years of life lost due to disability.

### DALY quantification

The data available were sufficient to model further necessary data and quantify DALYs attributable to mercury use in ASGM in Zimbabwe in 2004. The main analysis showed a total of 95,400 DALYs (8 DALYs/1,000 population) attributable to chronic mercury intoxication from occupational exposure (Table 7). Nearly the entire estimated disease burden is due to morbidity (Table 6). Considering the quantified EBD, the highest burden was found in male miners (>80% of the total DALYs), especially the age groups 9–14 (30% of total DALYs, 36% of total DALYs of males) and 15–24 years (40% of total DALYs, 49% of total DALYs of males), with a maximum of 28 DALYs/1,000 population amongst those aged 9–14. By contrast, the women affected most heavily were those aged 25–34, with 7 DALYs/1,000 population.

**Table 7:** **DALYs attributable to occupational mercury exposure from gold mining in Zimbabwe in 2004 by subgroup**

Age^*^(years)	Males			Females			Total		
	Population**	DALYs	DALYs per 1,000	Population**	DALYs	DALYs per 1,000	Population**	DALYs	DALYs per 1,000
**0-8**	1,540,000	1,200	<1	1,529,000	500	<1	3,069,000	1,700	<1
**9-14**	1,042,000	28,900	28	1,038,000	3,400	3	2,080,000	32,300	16
**15-24**	1,533,000	38,200	25	1,552,000	6,700	4	3,085,000	44,900	15
**25-34**	871,000	10,100	12	870,000	6,400	7	1,741,000	16,500	9
**35-41**	293,000	<100	<1	353,000	<100	<1	646,000	<100	<1
**≥42**	788,000	<100	<1	1,083,000	<100	<1	1,870,000	<100	<1
**Total**	6,067,000	78,400	13	6,425,000	17,000	3	12,492,000	95,400	8

All numbers are rounded.

The results of this analysis rest on the assumptions given in Table 2. Changing the assumptions will require changing the analysis.

*Age structure according to Table 4.

**Zimbabwe’s population in 2004; source: World population prospects by the United Nations [49].

DALYs: disability-adjusted life years.

### Scenario analysis

In addition to the assumptions made for the main analysis, Column E in Table 4 shows the minimum and maximum numbers assumed for ASG miners occupationally exposed to mercury in Zimbabwe which were included in the scenario analysis of prevalence (I). The lowest prevalence was calculated under the assumptions that 350,000 miners are exposed, that 50% of the adults are women, and that the proportion of children is 2%. These assumptions yielded an overall prevalence of 2% (Table 5). The highest prevalence of chronic mercury intoxication can be assumed if the number of occupationally exposed individuals is set to 500,000, with 2% children, and an adult sex distribution of 89% men and 11% women. If these assumptions hold, then 3% of the entire population of Zimbabwe is affected. This scenario range of prevalence (2-3%) was, together with other factors described below, included in the scenario analysis of DALY estimation (II).

Scenario analyses of DALY estimation (II) yielded a result variation of 6–12 DALYs/1,000 population (Additional file 1). Using an average maximum prevalence of 3% (rather than 2%), the total DALYs increase to 12 DALYs/1,000. The lowest DALY estimate of 6 DALYs/1,000 was made using an alternative DW (0.134 rather than 0.180). A variation of ±10% in the duration of disease yielded no significant differences. Including the mortality or relative risk (RR) of mortality instead of the case fatality in DisMod II, or omitting age weights and discount rates, led to only a slight difference in DALYs.

## Discussion

A total of 95,400 DALYs are estimated to be caused by chronic mercury intoxication due to artisanal small-scale gold mining (ASGM) in Zimbabwe in 2004 when using available and modeled data and applying the assumptions listed in Table 2. Table 8 shows the most important limitations and research needs related to the analysis.

**Table 8:** **Summary of the scope of the analysis: The most important limitations and research needs**

Scope of the analysis	Limitations	Research needs
**Research aim:** A first estimate of the DALYs of chronic mercury intoxication following occupational mercury use in ASGM on a national scale by using the methods applied in the GBD 2004 update and by using available data and information.	Data from earlier research projects are used while no special data survey was done to achieve the research aim.	Conduct surveys to improve the data basis for DALY estimates.
		Use the advanced methods to determine DALYs.
	Applying the methods of the GBD 2004 update because of the consistent reference year (2004) to enable comparisons while more advanced methods are available (GBD 2010 study [87]).	
		Conduct research to develop the DALY method (already started within the GBD 2010 study [87]).
	Specific limitations of the summary measure DALY (e.g., ethical concerns about using disability weights) are included in the analysis.	
**Subgroup of interest:** ASG miners (panners and smelter workers) with occupational exposure to mercury who work and live at a mining site.	Miners with acute, temporary or ending involvement in mining were not considered.	Conduct surveys to differentiate between subgroups of miners (duration of involvement, type of work, etc.) and to determine the burden of other exposed subgroups not or no longer actively involved in mining.
	No differentiation of burden between panners and smelter workers.	
	Other exposed subgroups (retired miners, residents at mining sites like the families of miners, etc.) were not considered.	
**Underlying sample:** 181 ASG miners in Kadoma; 91 controls from Chikwaka; surveyed in 2004 and 2006.	Small sample size.	Conduct comprehensive surveys at several mining sites in Zimbabwe using samples of adequate size.
	The sample included data from two projects conducted during two different survey periods.	
	All the information came from just one mining site in Zimbabwe (Kadoma).	
**Study population, country of interest, and reference year:** ASG miners in Zimbabwe in 2004.	Contradictory information on number, age, and sex distribution of ASG miners in Zimbabwe.	Verify the estimates of the number, age, and sex distribution of ASG miners in Zimbabwe.
	No information about the burden in other years (e.g., the current burden) and other countries (e.g., Colombia).	
		Quantify DALYs from other years and mining sites for comparison.
	No information about the burden when using mining methods and tools different from those in Kadoma.	
		Determine the health burden resulting from different mining methods and tools.
**Health outcome:** Chronic mercury intoxication as defined using the diagnostic tool developed by Drasch et al. [32]; assessed with a DW of 0.18; no remission; no mortality; no treatment.	Specific limitations of the diagnostic tool are included in the analysis (e.g., no correcting factor for health effects unrelated to mercury; all items of the medical score sum are weighted equally).	Conduct research to develop an improved diagnostic tool.
	There is no established and internationally accepted diagnosis for chronic mercury intoxication.	
		Establish an internationally accepted algorithm diagnosis for chronic mercury intoxication.
	There is no DW for chronic mercury intoxication; a provisional DW was used.	
		Derive a DW for chronic mercury intoxication.
	Remission and mortality data are scarce.	
		Conduct cohort studies to verify the assumptions of no remission and no mortality.
**Data modeling:** Lacking data were modeled using DisMod II.	It was necessary to model missing data.	Improve the data basis to allow analyses without modeled data.
	Specific limitations of DisMod II are included in the analysis.	

ASG: artisanal small-scale gold; ASGM: artisanal small-scale gold miners; DALYs: disability-adjusted life years; DisMod II: Disease Model, second version, a software tool developed by the World Health Organization; DW: Disability Weight; GBD: Global Burden of Disease and Injury.

Although several studies quantifying the BoD of (environmental) risk factors are available e.g., [88–93], none focused on a national burden of gold miners affected by mercury. Two other DALY quantifications including mercury are available. One focused on inorganic mercury exposure at toxic waste sites including but not limited to artisanal gold mining in India, Indonesia, and the Philippines. The burden of renal toxicity in the exposed population (2,122,200) was quantified, yielding a total of 83 DALYs (100% YLDs) [94]. The other quantification was based on methylmercury while including mild mental retardation. While the highest burden of the subgroups was seen in Brazilian fishing communities located near gold mining regions, a Canadian subgroup of sport fishers showed a burden of 7 DALYs/1,000 infants [27] comparable to the 8 DALYs/1,000 in Zimbabwe attributable to mercury use in gold mining (albeit not infants). Prüss-Üstün et al. [24] reviewed studies on the global burden of chemicals resulting in an attributable burden of nearly 6% of the estimated total global burden, while chemicals which had not been quantified, such as mercury, led to an underestimation.

The prevalence of chronic mercury intoxication according to the diagnosis used was calculated for samples from several countries. The total sample prevalence determined for miners (72%) was comparable to that found for ASGM sites in the Philippines (72% of miners) [32]. A rate of 24% was reported for amalgam smelter workers in Tanzania [23] and 55-62% in Indonesia [81, 95].

In other ASGM studies, human biomonitoring data and health effects were not combined to diagnose chronic mercury intoxication [21, 96], although it is recommended [13, 14]. The results of the medical score sum make it clear that the symptoms might also be present in controls not exposed to mercury. It can therefore be assumed that some health effects of the group exposed to mercury would be present even if they had not been exposed. This underlines the need for a combined diagnosis of chronic mercury intoxication (health and human biomonitoring data). It would be good if future studies derived a correction factor for excluding health effects not related to mercury.

Controls and miners differ significantly regarding lifestyle factors (alcohol, smoking, fish consumption), which is a limitation of the analysis because the factors could have biased the diagnosis of chronic mercury intoxication. The effects of alcohol consumption were previously analyzed in the data set from 2004. The mercury concentration and medical data of amalgam smelter workers with a high alcohol consumption were compared with the other smelter workers. No significant differences were found between the two subgroups except for the factor “memory problems” which could be biased by alcohol consumption [5]. Further detailed tests to explain the effects of possible confounders are recommended for future studies.

In previous ASGM studies (e.g., in the Zimbabwe data set from 2004 [5] which was used here), the correlation between mercury concentrations in human specimens and the items of the medical score sum were tested. Only a few correlations were found, which can be explained by the following: The blood-, urine-, and hair-mercury concentrations do not adequately reflect the concentrations in tissues like the brain. Furthermore, the exposure of gold miners is not limited to mercury vapor. Exposure to other forms of mercury with different toxicological effects is likely (e.g., methylmercury from eating fish). Since months or even years can pass before any symptoms appear, the correlation between the current concentration of mercury and current symptoms can be poor [5, 14, 32, 81].

This last explanation can also be applied to the following result. A subclinical intoxication was diagnosed in the case of six miners (Additional file 4). Their medical score sum was low but the concentration of mercury was high (>BAT). Exceeding the German exposure limit values (HBM I, HBM II, BAT) makes health effects possible, but does not predict them. Unlike the subclinical cases, 10 miners showed a high medical score sum but a mercury concentration between HBM I and HBM II. Thus, differences in individual susceptibility are conspicuous and should be analyzed in detail in future studies.

It can also be useful to weight the items used for diagnosis differently, as discussed previously [14]. However, including different weights would require ranking items, which would in turn require further assumptions.

In addition to applying other diagnoses, prevalence can differ between regions because of mining methods (e.g., whole ore amalgamation or amalgamation of concentrates) or tools (e.g., different mills) [97], probably also because of cultural differences such as misconceptions about handling mercury [4, 9, 50]. There is a research need for comparable EBD estimates from other mining regions which might assess possible different impacts on the health of the miners (Table 8).

Exposure to mercury in Kadoma district becomes apparent when considering human biomonitoring concentrations (e.g., median urine concentration of 26 μg/l and 26 μg/g creatinine; Additional file 3). In this analysis, urine-, blood-, and hair-mercury concentrations were used because of their different indications regarding the body burden: Inorganic mercury raises concentrations in urine more than it does in blood, whereas organic mercury raises blood concentrations [34]. Hence, exposure to mainly inorganic mercury (e.g., exposure due to vapor) can be assumed for this cohort.

Analyses of the HBM concentrations of the 2004 and 2006 project have been done before, albeit separately and with other subgroups. A median mercury concentration of 37 μg/l urine (26 μg/g creatinine) was reported for male and female miners aged 15–60 in 2004, and 5 μg/l urine (4 μg/g creatinine) for female miners of the same age in 2006. Compared to ASGM areas in Indonesia, Mongolia, the Philippines, and Tanzania, Zimbabwe (2004) showed the highest median and the second highest maximum urine mercury concentrations (1,530 μg/l) after Kalimantan in Indonesia (maximum 5,240 μg/l) [48].

Despite their young age, children in Indonesia and Zimbabwe living in contaminated areas and working with mercury showed 9 resp. 27 μg/g creatinine in urine [12]. A sample of 46 women of child-bearing age from Indonesia, Tanzania, and Zimbabwe showed median mercury concentration in breast milk of 2 μg/l [98].

Another study in Zimbabwe reported high blood-mercury concentrations, defined as >0.05 mg/l (corresponding to 50 μg/l) in 17% of a sample of 66 adult miners [96]. Miner cohorts in South West Ghana (mean of 1 μg Hg/l urine) [99], Peru (mean/median of 9 μg Hg/g creatinine) [100], Suriname (mean of 30 μg Hg/g creatinine) [101], Thailand (mean of >35 μg Hg/g creatinine) [102], and northern Tanzania (mean of nearly 40 μg Hg/g creatinine) [103] showed lower biomonitoring concentrations in miners compared to the results of this study, while higher median urine mercury concentrations (44 μg/l) were reported for amalgam smelting miners in the Upper East Region in Ghana [104]. In Venezuela and Burkina Faso the average urine mercury concentrations reached nearly 105 μg/g creatinine [22] resp. 195 μg/g creatinine [21].

Unspecific health effects possibly associated with exposure to mercury were seen more often in exposed individuals than in controls. Nearly 90% of gold miners in Zimbabwe, but <60% of controls reached a medical score sum of 5 or more. By contrast, in Burkina Faso half the gold miners showed at least five health symptoms associated with mercury [21].

### Subgroup results and excluded groups at risk

The results show the highest burden in the youngest age group and a decreasing burden in the oldest (Table 7). A high mercury burden for children and infants as well as breast-feeding mothers are especially alarming [105] because mercury damages developing brain tissue and induces lifelong adverse effects [9, 12]. The generally young population of Zimbabwe [49], the highest prevalence of chronic mercury intoxication amongst miners aged 15–24 years (Table 7) and the decreasing subgroup size by age caused by retirement from mining due to poor health might explain the distribution of the disease burden. Zimbabweans who stop mining often leave the site and return to their home region [52], which makes it difficult to analyze the burden in this particular subgroup and collect data on remission and mortality. An underestimation can therefore be assumed due to a high turnover rate. Individuals with mercury intoxication leave the mining site and so are not included in study samples (and hence not counted), while healthy people without intoxication begin working at the mine.

Environmentally exposed residents in ASGM areas also show considerable exposure and health effects due to mercury [7, 20, 23, 100, 106, 107]. It can be assumed that two million inhabitants are exposed in ASGM areas in Zimbabwe, consisting of the miners’ families and people with other jobs such as truck drivers and cooks [50, 62, 108–111]. They are also exposed because of the unregulated use of mercury, inadequate separation of workplace and home (e.g., storing clothes worn for work at home; see above), and extensive contamination of entire areas [5, 12, 32].

Although it was possible to include only one – the most important – subgroup in the analysis, a substantial additional burden can be assumed from occupationally unexposed inhabitants and retired miners, which has not yet been quantified. It is therefore necessary to conduct comprehensive, detailed studies on all relevant subgroups for an accurate picture of the entire burden caused by mercury exposure from gold mining (Table 8). However, the used diagnostic tool needs to be improved for analyzing retired miners, because the half-life of mercury in the used specimens is short in comparison to the half-life of mercury in body tissues. A negative diagnosis based on low mercury concentrations in urine, blood, or hair of no longer exposed individuals could be the result, although health effects and enhanced concentrations in body tissues might be present [78].

### Number and distribution of individuals at risk

Comprehensive data on the situation and the extent of ASGM are largely lacking [6, 7], and the available estimates contain uncertainties and limitations. In addition to the informal nature of ASGM, fluctuations in the number of seasonal and occasional workers reduce the quality of estimates [54]. Sources often contain less information on the original sources, the estimation basis, or the exact year of the estimates. Thus, references from the late 20^th^ and the early 21^st^ century were taken as an approximation for 2004. In the literature, the number of miners in Zimbabwe during this time period is estimated at 50,000-500,000 and over, with 350,000 cited the most often [8, 9, 50, 51, 53–56, 108, 110, 112–115]. The higher estimates are mentioned in the more recent publications, which might reflect the quick spread of ASGM [50]. These estimates are based on the number of miners per kilometer of river [8], mining, minerals and sustainable development (MMSD) global and country artisanal small-scale mining (ASM) reports [51], governmental representatives, non-governmental organizations, and mining bodies which took part in conferences on this subject [54] as well as questionnaires, technical journals, and UN and World Bank agencies [9]. Some sources refer to ASM in general but not explicitly to gold mining, even though over 90% of ASM in Zimbabwe is for gold [50].

The miners in the sample consisted of panners and smelter workers. Exposure is highest amongst miners involved in amalgam smelting, resulting in a higher mercury body burden, worse health effects, and a higher percentage of intoxications [5]. Since the proportion of panners and smelter workers in Zimbabwe is unknown, no extrapolation is currently possible. It is therefore essential that estimates of the distribution of panners and smelter workers as well as a separate analysis of both groups be done in the future (Table 8).

### Health data

Comprehensive information on diseases occurring in ASGM areas is largely lacking, and health effects attributable to mercury can be masked by the generally poor health conditions at the mining sites and the absence of facilities for medical diagnosis [5, 6]. Hence, in addition to data from field projects, information from the literature and data modeling were necessary for the DALY quantification.

As in the GBD 2004, DisMod II was used to model the data, which ensures comparability of results. While data modeling itself is an approximation, the underlying prevalence data also contained uncertainties. Prevalence was determined using a restricted number of age groups and based on a relatively small sample size (n = 181) surveyed cross-sectionally in one region (Kadoma district) in two years (2004 and 2006). Combining two samples from different years could include changes over time. Furthermore, the special focus of the second (2006) cohort on women of child-bearing age is a limiting factor. Data quality might be lowered by information from project participants which cannot be verified. Also, blinded testing was not possible because the exposed group was located more than 100 km from the controls [5].

Causality of the association between exposure and disease was confirmed by using a control group and detecting mercury in human biomonitoring samples in addition to collecting health data. Nevertheless, comprehensive, longitudinal cohort studies observing health effects and mercury in human biomonitors are urgently needed (Table 8).

The scarcity of data on remission, and particularly on duration of disease, is a common limitation in BoD assessments because cohort studies are rare [83]. While duration was modeled using DisMod II, zero remission was assumed in this analysis based on findings reported in the literature. Nevertheless, not including remission cases is in keeping with the assumption that cases identified by the underlying diagnosis are severe and show irreversible damage (Table 2, Assumption j). Without remission, the disease is likely to be of long duration (Table 6).

Mortality data also are scarce. One study focusing on the mortality of workers chronically exposed to elemental mercury vapor did not find a higher mortality compared to controls [62]. Mortality attributable to high acute mercury exposure was confirmed [33, 116], as were higher suicide rates amongst people exposed to mercury, these due to changes in personality [19, 117]. Furthermore, there is a possible association with increased mortality from cardiovascular disease as a result of long-term exposure in European mercury mines [118]. Both demonstrated mortality causes might be sequelae of mercury exposure, and its burden needs to be quantified separately rather than compounded with the burden of chronic mercury intoxication. Hence, no case fatality was assumed in this analysis (Table 2, Assumption i; although negligible four cases were modeled based on the data given) because every other assumption would be inferred without underlying epidemiological information.

### Analyses of competing scenarios

EBD estimates contain uncertainties, limitations, and assumptions [119]. Besides the transparent description, competing information is included in the scenario analyses. Age weights and discount rates were used in this analysis to allow comparison with the GBD 2004 update. Nevertheless, both aspects are controversial [86], which is why their influence should be analyzed. When omitting both factors, which was the approach used for the GBD 2010 [87], the total DALYs in the main analysis rose from 8 to 9 DALYs/1,000 population. An appropriate DW is more sensitive when determining DALYs for chronic mercury intoxication because of its relevance for morbidity. For this preliminary analysis, a DW of a comparable condition was used, as was done before e.g., [27, 120, 121]. Alcoholism is considered a confounding variable with mercury studies because it causes similar symptoms [7, 13, 14, 32, 61], which might justify using its DW for an approximation. Nevertheless, there is an urgent need to verify the DW used while estimating a specific DW for chronic mercury intoxication in future studies (Table 8).

The scenario analyses increased the meaningfulness of the results by yielding a scenario range (6–12 DALYs/1,000 population) which was not significant to the central message about the public health impact of chronic mercury intoxication. Nevertheless, it is important to consider that one parameter was varied per scenario analysis while the effects of varying more than one parameter at a time were not examined. Further scenario analyses with hypothetical assumptions could be conducted, although solid evidence would certainly be preferable. By assuming an increasing relevance of ASGM caused by unemployment and rising gold prices [54, 97], it is likely for DALYs to be distinctly higher if no comprehensive interventions are implemented.

### Comparing with Zimbabwe’s leading causes of disease burden

When accepting the assumptions determined and recognizing the caveats originated from the limited data available, a number of 6–12 DALYs per 1,000 population can be assumed as result of the preliminary estimates. Putting the results of the current analysis in context with 132 categories of disease and injury in the GBD 2004 update [31], chronic mercury intoxication due to the use of mercury in ASGM would be amongst the top 20 causes of BoD in Zimbabwe in 2004. The total burden in Zimbabwe in 2004 as determined by the GBD was 680 DALYs/1,000 population [31, 122]. When integrating the preliminary estimate of DALYs due to chronic mercury intoxication, it would be as high as 1% of the total DALYs. By comparison, 1% of the global BoD is estimated to be attributable to lead-induced loss of IQ points, anemia, gastrointestinal symptoms in children, and cardiovascular diseases in adults [123]. Less than 2 DALYs/1,000 population in Zimbabwe in 2004 were assumed to be attributable to alcohol use disorders [122]. In this analysis, their severity (DW) was assumed to be comparable to chronic mercury intoxication.

When comparing the preliminary DALYs obtained in the current analysis, the public health relevance of mercury in gold mining is underlined.

### Usefulness of DALY estimates

The WHO notes that policy makers seem to be more acutely aware of mortality statistics than of non-fatal disabilities [124]. In the case of chronic mercury intoxication, there is no mortality case reported which probably explains the low priority given this condition. A lack of data could be a reason for the continued use of mercury in gold mining and a lack of comprehensive exposure reduction in ASGM so far. Summary measures like the DALY, which combine mortality and morbidity, have vital advantages, especially with non-fatal diseases, as well as with diseases where the morbidity burden is essential [124]. The analysis emphasizes the benefits of the EBD method for public health problems which are not of primary relevance. Nevertheless, the results of this EBD analysis can only be accepted if the underlying assumptions (Table 2) are accepted, while long-term cohort studies are urgently needed to corroborate these assumptions (Table 8).

## Conclusions

The health burden from occupational mercury use in ASGM is likely to be an important public health hazard in Zimbabwe, what is the result of this analysis using the scarce data available. Based on our analysis and underlying assumptions, chronic mercury intoxication is likely to have been one of the top 20 leading causes of disability in Zimbabwe in 2004, with more than 95,000 DALYs. This study is a preliminary quantification because of the limited evidence and the necessity to model data and determine assumptions at almost all stages of the analysis. Research is necessary to define an internationally accepted diagnosis of chronic mercury intoxication, to fully understand the progress of the disease (remission, duration, mortality), to determine its severity in comparison to other diseases (DW), and to verify the number of the exposed and diseased population. More detailed and complete data will be needed to verify and provide more accurate estimates.

The advantages of the EBD concept are highlighted by revealing important insights into the burden of a hitherto neglected but important public health issue. This analysis has shown that even if data are incomplete, information can be used to classify the public health relevance of less well-known diseases and conditions.

Following the preliminary analysis, chronic mercury intoxication is likely to be an important health issue in gold mining communities. The assumed high burden amongst young miners in this analysis is particularly alarming. These findings should be used to raise awareness and encourage the introduction of comprehensive initiatives to reduce this avoidable health burden and improve the health situation in ASGM areas in Zimbabwe and elsewhere.

## Electronic supplementary material

Additional file 1:Parameter variation in the scenario analysis.(PDF 19 KB)

Additional file 2:Summary of sociodemographic characteristics.(PDF 18 KB)

Additional file 3:Human biomonitoring concentrations and categorization into exposure limit values.(PDF 26 KB)

Additional file 4:Detailed analyses of the health effects in the control and the exposed group.(PDF 63 KB)

## Abbreviations

ASG: Artisanal small-scale gold
ASM: Artisanal small-scale mining
ASGM: Artisanal small-scale gold mining
BAT: Biological workplace tolerance level (Biologischer Arbeitsplatztoleranzwert)
BoD: Burden of disease
CRA: Comparative risk assessment
DALY: Disability-adjusted life year
DisMod II: Disease Model, second version, software tool developed by the World Health Organization
DW: Disability weight
EBD: Environmental burden of disease
GBD: Global Burden of Disease and Injury
GEF: Global Environment Facility
GMP: Global Mercury Project
HBM: Human Biomonitoring
Hg: Mercury
LMU: University of Munich
MMSD: Mining, minerals and sustainable development
UN: United Nations
UNDP: United Nations Development Programme
UNEP: United Nations Environment Programme
UNIDO: United Nations Industrial Development Organization
WHO: World Health Organization
YLD: Years lived with disability
YLL: Years of life lost due to premature mortality.
