# Supplementary material for: The burden of chronic mercury intoxication in artisanal small-scale gold mining in Zimbabwe: data availability and preliminary estimates
Source: Environ Health. 2014 Dec 13;13:111. doi: 10.1186/1476-069X-13-111 (PMC4290131; doi:10.1186/1476-069X-13-111)
Supplement: Supplementary file 1 — Additional file 1:Parameter variation in the scenario analysis.(PDF 19 KB) [file 12940_2014_818_MOESM1_ESM.pdf]

Additional files

Additional file 1 – Parameter variation in the scenario analysis

Scenario	Incidence (%)*	DW#	Case fatality	Age weights **	Discount rate**	Other variation	DALYs / 1,000 ***
Main analysis	2.12	0.180	0	used	used	-	8
Scenario analysis							
1	**1.85**	0.180	0	used	used	-	7
2	**3.37**	0.180	0	used	used	-	12
3	2.12	0.180	**-**	used	used	**Mortality = 0**	9
4	2.12	0.180	**-**	used	used	**RR mortality = 1**	8
5	2.12	0.180	0	used	used	**+10% of modeled duration of disease**	8
6	2.12	0.180	0	used	used	**-10% of modeled duration of disease**	7
7	2.12	0.180	0	**omitted**	**omitted**	-	9
8	2.12	**0.134**	0	used	used	-	6

* Modeled using DisMod II. The scenario range of the incidence included here is the result of the scenario analysis of the underlying prevalence which was fed into DisMod II.

** Age weights: non-uniform; discount rate: 3% [1, 2].

*** Numbers are rounded.

DW for alcohol dependence: 0.180 [3]; DW for alcohol use disorders: 0.134 [1].

Highlighted in bold: varied parameter

DALYs: disability-adjusted life years; DW: disability weight; RR mortality: relative risk of mortality

References

1. WHO (World Health Organization): **The Global Burden of Disease: 2004 update**. Geneva; 2008.
2. Pruss-Ustun A, Mathers C, Corvalan C, Woodward A: **Introduction and methods: Assessing the environmental burden of disease at national and local levels. Environmental Burden of Disease Series, No. 1**. Geneva: WHO (World Health Organization); 2003.
3. Murray CJ, Lopez AD: **The global burden of disease: a comprehensive assessment of mortality and disability from diseases, injuries, and risk factors in 1990 and projected to 2020**. Boston: Harvard School of Public Health on behalf of the WHO (World Health Organization) and the World Bank; 1996.
