# Supplementary material for: The burden of chronic mercury intoxication in artisanal small-scale gold mining in Zimbabwe: data availability and preliminary estimates
Source: Environ Health. 2014 Dec 13;13:111. doi: 10.1186/1476-069X-13-111 (PMC4290131; doi:10.1186/1476-069X-13-111)
Supplement: Supplementary file 2 — Additional file 2:Summary of sociodemographic characteristics.(PDF 18 KB) [file 12940_2014_818_MOESM2_ESM.pdf]

Additional files

Additional file 2 – Summary of sociodemographic characteristics

Characteristic	Controls		Occupationally exposed	
	Total (percentage)	Male/female (percentage)	Total (percentage)	Male/female (percentage)
Total sample	91 (100%)	24/67 (26%/74%)	181 (100%)	122/59 (67%/33%)
2004 survey	48 (53%)	24/24 (50%/50%)	155 (86%)	122/33 (79%/21%)
2006 survey	43 (47%)	0/43 (0%/100%)	26 (14%)	0/26 (0%/100%)

Characteristic	Controls				Occupationally exposed			
	Range (min-max)	Mean	Median	95th percentile	Range (min-max)	Mean	Median	95th percentile
Age distribution (years)	11-59	24	23	39	9-75	27	25	46
Years living in the area	0-59	11	6	35	0-59	9	5	27
Years of exposure to mercury	0	0	0	0	1-23	4	3	12

Data sources: The data were taken from the Global Mercury Project (GMP) conducted by UNIDO in Zimbabwe in 2004 [1, 2], and from a health and biomonitoring project focusing on women of child-bearing age and their infants conducted by the University of Munich (LMU; Germany) in Zimbabwe in 2006 [3].

References

1. Bose-O'Reilly S, Dahlmann F, Lettmeier B, Drasch G: **Removal of barriers to the introduction of cleaner artisanal gold mining and extraction technologie in Kadoma, Zimbabwe – Final Report, Part B Health Assessment**. Orléans: Bureau de Recherches Géologiques et Minières (BRGM); 2004.
2. Bose-O'Reilly S, Lettmeier B, Gothe RM, Beinhoff C, Siebert U, Drasch G: **Mercury as a serious health hazard for children in gold mining areas**. *Environ Res* 2008, **107**(1):89-97.
3. Baeuml J, Bose-O'Reilly S, Matteucci Gothe R, Lettmeier B, Roider G, Drasch G, Siebert U: **Human Biomonitoring Data from Mercury Exposed Miners in Six Artisanal Small-Scale Gold Mining Areas in Asia and Africa**. *Minerals* 2011, **1**:122-143.
