# Supplementary material for: The burden of chronic mercury intoxication in artisanal small-scale gold mining in Zimbabwe: data availability and preliminary estimates
Source: Environ Health. 2014 Dec 13;13:111. doi: 10.1186/1476-069X-13-111 (PMC4290131; doi:10.1186/1476-069X-13-111)
Supplement: Supplementary file 3 — Additional file 3:Human biomonitoring concentrations and categorization into exposure limit values.(PDF 26 KB) [file 12940_2014_818_MOESM3_ESM.pdf]

Additional files

Additional file 3 – Human biomonitoring concentrations and categorization into exposure limit values

**Human biomonitoring**	**Subgroups**	
	**Controls**	**Occupationally exposed**
Hg in urine*	n=91	n=181
Mean (µg/l)	0.3	91.3
Median (µg/l)	<LOD	26.1
Range (min.-max.) (µg/l)	<LOD - 8.8	<LOD - 1,530.3
95^{th}percentile (µg/l)	1.3	376.9
Hg in urine, exposure limit values (p<0.001)*		
Below HBM I	90 (99%)	38 (21%)
Between HBM I and HBM II	1 (1%)	49 (27%)
Between HBM II and BAT	0 (0%)	7 (4%)
Above BAT	0 (0%)	87 (48%)
Hg in urine corrected for creatinine (crea.)*	n=80	n=181
Mean (µg/g crea.)	0.2	58.3
Median (µg/g crea.)	<LOD	25.8
Range (min.-max.) (µg/g crea.)	<LOD - 3.6	<LOD - 666.9
95th percentile (µg/g crea.)	1.1	214.5
Hg in urine (crea.), exposure limit values (p<0.001)*		
Below HBM I	80 (100%)	32 (18%)
Between HBM I and HBM II	0 (0%)	47 (26%)
Between HBM II and BAT	0 (0%)	9 (5%)
Above BAT	0 (0%)	93 (51%)
Hg in blood*	n=48	n=152
Mean (µg/l)	0.5	18.5
Median (µg/l)	0.4	11.4
Range (min.-max.) (µg/l)	<LOD - 1.9	<LOD - 100.8
95^{th}percentile (µg/l)	1.7	58.0
Hg in blood, exposure limit values (p<0.001)*		
Below HBM I	48 (100%)	34 (22%)
Between HBM I and HBM II	0 (0%)	56 (37%)
Between HBM II and BAT	0 (0%)	52 (34%)
Above BAT	0 (0%)	10 (7%)
Hg in hair*	n=79	n=158
Mean (µg/l)	0.2	8.9
Median (µg/l)	0.1	3.3
Range (min.-max.) (µg/l)	0.1 - 3.3	0.3 - 112.2
95^{th}percentile (µg/l)	0.3	34.0
Hg in hair, exposure limit values (p<0.001)*		
Below HBM I	77 (97%)	18 (11%)
Between HBM I and HBM II	2 (3%)	84 (53%)
Between HBM II and BAT	0 (0%)	56 (35%)

Data sources: The data were taken from the Global Mercury Project (GMP) conducted by UNIDO in Zimbabwe in 2004 [1, 2], and from a health and biomonitoring project focusing on women of child-bearing age and their infants conducted by the University of Munich (LMU; Germany) in Zimbabwe in 2006 [3]. HBM I and II for blood and urine were taken from the Commission for Human Biomonitoring of the German Federal Environment Agency [4-6]; BAT for blood and urine were taken from the German Research Foundation [7]. HBM I for hair was derived by Drasch et al. [8] from the U.S. EPA benchmark limit [9]. HBM II for hair was derived by Drasch et al. [8] from the HBM II value for blood [4-6] together with results from the Seychelles study [10].

*LOD: The limit for determining total mercury in urine and blood was 0.20 µg/l, and 0.02 µg/g in hair related to a 100 mg hair sample. Values below the detection threshold were included in the statistical analyses with one-half the detection threshold.

** A sum of less than 100% is due to rounding.

References

1. Bose-O'Reilly S, Dahlmann F, Lettmeier B, Drasch G: **Removal of barriers to the introduction of cleaner artisanal gold mining and extraction technologie in Kadoma, Zimbabwe – Final Report, Part B Health Assessment**. Orléans: Bureau de Recherches Géologiques et Minières (BRGM); 2004.
2. Bose-O'Reilly S, Lettmeier B, Gothe RM, Beinhoff C, Siebert U, Drasch G: **Mercury as a serious health hazard for children in gold mining areas**. *Environ Res* 2008, **107**(1):89-97.
3. Baeuml J, Bose-O'Reilly S, Matteucci Gothe R, Lettmeier B, Roider G, Drasch G, Siebert U: **Human Biomonitoring Data from Mercury Exposed Miners in Six Artisanal Small-Scale Gold Mining Areas in Asia and Africa**. *Minerals* 2011, **1**:122-143.
4. German Human Biomonitoring (HBM) Commission of the Federal Environment Agency (UBA): **Stoffmonographie Quecksilber – Referenz- und Human-Biomonitoring-(HBM)-Werte**. *Bundesgesundheitsbl – Gesundheitsforsch – Gesundheitsschutz* 1999, **42**(6):522-532 [German].
5. German Human Biomonitoring (HBM) Commission of the Federal Environment Agency (UBA): **Addendum zur „Stoffmonographie Quecksilber – Referenz- und Human-Biomonitoring-Werte" der Kommission Human-Biomonitoring des Umweltbundesamtes**. *Bundesgesundheitsbl – Gesundheitsforsch – Gesundheitsschutz* 2009, **52**:1228-1234 [German].
6. Schulz C, Wilhelm M, Heudorf U, Kolossa-Gehring M: **Reprint of "Update of the reference and HBM values derived by the German Human Biomonitoring Commission"**. *Int J Hyg Environ Health* 2012, **215**(2):150-158.
7. DFG (Deutsche Forschungsgemeinschaft): **MAK- und BAT-Werte-Liste**. Weinheim: Wiley VCH; 2009 [German].
8. Drasch G, Bose-O'Reilly S, Beinhoff C, Roider G, Maydl S: **The Mt. Diwata study on the Philippines 1999 - assessing mercury intoxication of the population by small scale gold mining**. *Sci Total Environ* 2001, **267**:151-168.
9. U.S. EPA (United States Environmental Protection Agency): **Mercury Study Report to Congress. Volume V: Health Effects of Mercury and Mercury Compounds**, vol. EPA-452/R-97-003. Washington D.C.; 1997.
10. Davidson PW, Myers GJ, Cox C, Axtell C, Shamlaye C, Sloane-Reeves J, Cernichiari E, Needham L, Choi A, Wang Y *et al*: **Effects of prenatal and postnatal methylmercury exposure from fish consumption on neurodevelopment: outcomes at 66 months of age in the Seychelles Child Development Study**. *JAMA* 1998, **280**(8):701-707.
