# Supplementary material for: The burden of chronic mercury intoxication in artisanal small-scale gold mining in Zimbabwe: data availability and preliminary estimates
Source: Environ Health. 2014 Dec 13;13:111. doi: 10.1186/1476-069X-13-111 (PMC4290131; doi:10.1186/1476-069X-13-111)
Supplement: Supplementary file 4 — Additional file 4:Detailed analyses of the health effects in the control and the exposed group.(PDF 63 KB) [file 12940_2014_818_MOESM4_ESM.pdf]

Additional files

Additional file 4 – Detailed analyses of the health effects in the control and the exposed group

Additional table 1 - Positive findings: Anamnestic data and score by subgroups

Subgroup / Anamnesticdata	Control group	Occupationally exposed	Missing	p value
Metallic taste	3.3% (n= 3)	18.8% (n= 34)	0	<0.001
Excessive salivation	6.7% (n= 6)	13.3% (n= 24)	2	0.109
Tremor at work	0% (n= 0)	13.3% (n= 24)	2	<0.001
Sleeping problems at night	11% (n= 10)	8.8% (n= 16)	0	0.569
Health problems worsened since mercury exposed	/	2.8% (n= 5)	0	/
**Anamnestic Score**				
0	82.4% (n= 75)	59.1% (n=107)	/	0.003
1	14.3% (n= 13)	28.7% (n= 52)		
2	3.3% (n= 3)	8.8% (n= 16)		
3	0% (n=0)	2.8% (n= 5)		
4	0% (n=0)	0.6% (n=1)		
Data sources: The data were taken from the Global Mercury Project (GMP) conducted by UNIDO in Zimbabwe in 2004 [1, 2], and from a health and biomonitoring project focusing on women of child-bearing age and their infants conducted by the University of Munich (LMU; Germany) in Zimbabwe in 2006 [3].				

Additional table 2 - Positive findings: Clinical data and score by subgroups

Subgroup / Clinical data	Control group	Occupationally exposed	Missing	p value
Bluish coloration of the gingiva	7.7% (n= 7)	27.6 (n= 50)	0	<0.001
Ataxia of gait	18.7% (n= 17)	34.8% (n= 63)	0	0.006
Finger-to-nose tremor	0% (n=0)	5.0% (n= 9)	3	0.030
Dysdiadochokinesia	11.1% (n= 10)	28.5% (n= 51)	3	0.001
Heel-to-knee ataxia	5.7% (n= 5)	12.2% (n= 22)	4	0.095
Heel-to-knee tremor	0.0% (n= 0)	0.0% (n= 0)	4	/
Mento-labial reflex	23.1% (n= 21)	27.1% (n= 49)	0	0.477
Proteinuria	18.9% (n= 17)	10.6% (n= 19)	2	0.058
**Clinical Score**				
0	42.9% (n= 39)	24.3% (n= 44)	/	<0.001
1	33.0% (n=30)	33.7% (n= 61)		
2	20.9% (n= 19)	19.9% (n= 36)		
3	3.3% (n= 3)	16.6% (n= 30)		
4	0% (n=0)	5.5% (n= 10)		
Data sources: The data were taken from the Global Mercury Project (GMP) conducted by UNIDO in Zimbabwe in 2004 [1, 2], and from a health and biomonitoring project focusing on women of child-bearing age and their infants conducted by the University of Munich (LMU; Germany) in Zimbabwe in 2006 [3].				

Additional table 3 - Positive findings: Neuropsychological results and score by subgroups

Subgroup / Neuro-psycho-logical tests	Score *	Control group	Occupationally exposed	Missing	Chi^2
Memory test	1	60.4% (n= 29)	57.5% (n= 88)	71	0.106
	2	22.9% (n= 11)	34.6% (n= 53)		
Matchbox test	1	47.8% (n= 43)	37.8% (n= 68)	2	0.287
	2	43.3% (n= 39)	52.2% (n= 94)		
Frostig test	1	50% (n= 24)	58.4% (n= 90)	70	0.006
	2	47.9% (n= 23)	27.3 (n= 42)		
Pencil tapping test	1	28.9% (n= 26)	11.7% (n= 21)	2	<0.001
	2	60.0% (n= 54)	87.2% (n= 157)		
* 1= restricted performance; 2= bad performance					
**Neuropsychological Score**					
0		2.2% (n= 2)	0.6% (n= 0)	1	<0.001
1		4.4% (n= 4)	0.0% (n= 0)		
2		17.6% (n= 16)	5.0% (n= 9)		
3		19.8% (n= 18)	6.1% (n= 11)		
4		12.1% (n= 11)	13.3% (n= 24)		
5		15.4% (n= 14)	28.3% (n= 51)		
6		16.5% (n= 15)	23.3% (n= 42)		
7		6.6% (n= 6)	17.2% (n= 31)		
8		5.5% (n= 5)	6.1% (n= 11)		
Data sources: The data were taken from the Global Mercury Project (GMP) conducted by UNIDO in Zimbabwe in 2004 [1, 2], and from a health and biomonitoring project focusing on women of child-bearing age and their infants conducted by the University of Munich (LMU; Germany) in Zimbabwe in 2006 [3].					

Additional table 4 - Summary of the medical score sum by subgroups

**Medical score sum** (score points)	**Subgroups**		**p value**
	**Controls (%)**	**Occupationally exposed (%)**	
Low (0-4)	37 (41%)	20 (11%)	<0.001
Medium (5-9)	52 (57%)	130 (72%)	
High (10-21)	2 (2%)	31 (17%)	
Data sources: The data were taken from the Global Mercury Project (GMP) conducted by UNIDO in Zimbabwe in 2004 [1, 2], and from a health and biomonitoring project focusing on women of child-bearing age and their infants conducted by the University of Munich (LMU; Germany) in Zimbabwe in 2006 [33].			

Additional table 5 - Number of miners and controls in diagnostic groups

**Exposure limit values [b]**		**Medical score sum [c]**		
		Low (0-4)	Medium (5-9)	High (10-21)
Hg in all biomonitors	<HBM I	No intoxication<br>C: 41 % (n= 37)<br>M: 2% (n= 3)	No intoxication<br>C: 55% (n= 50)<br>M: 4% (n= 7)	No intoxication<br>C: 2% (n= 2)<br>M: 0% (n= 0)
Hg in at least one biomonitor	>HBM I, <HMB II	No intoxication<br>C: 0% (n= 0)<br>M: 6% (n= 11)	No intoxication<br>C: 2% (n= 2)<br>M: 16% (n= 29)	**INTOXICATION**<br>**C: 0% (n= 0)**<br>**M: 6% (n= 10)**
	>HBM II, <BAT	No intoxication<br>C: 0% (n= 0)<br>M: 0% (n= 0)	**INTOXICATION**<br>**C: 0% (n= 0)**<br>**M: 8% (n= 14)**	**INTOXICATION**<br>**C: 0% (n= 0)**<br>**M: 2% (n= 3)**
	>BAT	**INTOXICATION**<br>**C:** 0% (n= 0)<br>**M: 3% (n= 6)**	**INTOXICATION**<br>**C: 0% (n= 0)**<br>**M: 44% (n= 80)**	**INTOXICATION**<br>**C: 0% (n= 0)**<br>**M: 10% (n= 18)**

Percentages are rounded.

BA: Biologischer Arbeitsplatztoleranzwert [maximum acceptable concentration at the workplace]; C: controls; M: miners; HBM I and II: Human Biomonitoring values from the Human Biomonitoring Commission of the Federal Environmental Agency; n: number.

Data sources: The data were taken from the Global Mercury Project (GMP) conducted by UNIDO in Zimbabwe in 2004 [1, 2], and from a health and biomonitoring project focusing on women of child-bearing age and their infants conducted by the University of Munich (LMU; Germany) in Zimbabwe in 2006 [3].

(light grey)	None of the cases in the subgroup belong in this diagnostic category.
(dark grey)	1-9% of the cases in the subgroup belong in this diagnostic category.
(black)	≥10% of the cases in the subgroup belong in this diagnostic category.

Additional figure 1 - Visualization of miners and controls in diagnostic groups

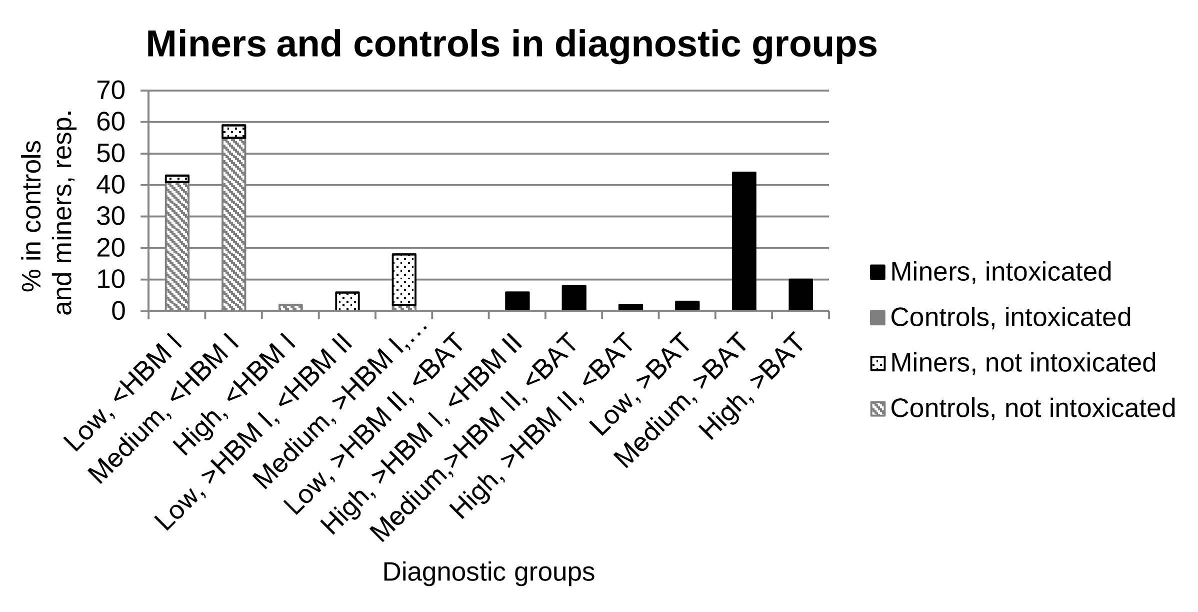

References

1. Bose-O'Reilly S, Dahlmann F, Lettmeier B, Drasch G: **Removal of barriers to the introduction of cleaner artisanal gold mining and extraction technologie in Kadoma, Zimbabwe – Final Report, Part B Health Assessment**. Orléans: Bureau de Recherches Géologiques et Minières (BRGM); 2004.
2. Bose-O'Reilly S, Lettmeier B, Gothe RM, Beinhoff C, Siebert U, Drasch G: **Mercury as a serious health hazard for children in gold mining areas**. *Environ Res* 2008, **107**(1):89-97.
3. Baeuml J, Bose-O'Reilly S, Matteucci Gothe R, Lettmeier B, Roider G, Drasch G, Siebert U: **Human Biomonitoring Data from Mercury Exposed Miners in Six Artisanal Small-Scale Gold Mining Areas in Asia and Africa**. *Minerals* 2011, **1**:122-143.
